# Responsibility Gaps and Black Box Healthcare AI: Shared Responsibilization as a Solution

**DOI:** 10.1007/s44206-023-00073-z

**Published:** 2023-11-16

**Authors:** Benjamin H. Lang, Sven Nyholm, Jennifer Blumenthal-Barby

**Affiliations:** 1 University of Oxford, Oxford, UK; 2 Baylor College of Medicine, Houston, TX, USA; 3 LMU Munich, Munich, Germany; 4 Munich Center for Machine Learning, Munich, Germany

**Keywords:** Artificial intelligence, Responsibilization, Black box healthcare AI, Responsibility gaps, Shared responsibility

## Abstract

As sophisticated artificial intelligence software becomes more ubiquitously and more intimately integrated within domains of traditionally human endeavor, many are raising questions over how responsibility (be it moral, legal, or causal) can be understood for an AI’s actions or influence on an outcome. So called “responsibility gaps” occur whenever there exists an apparent chasm in the ordinary attribution of moral blame or responsibility when an AI automates physical or cognitive labor otherwise performed by human beings and commits an error. Healthcare administration is an industry ripe for responsibility gaps produced by these kinds of AI. The moral stakes of healthcare are often life and death, and the demand for reducing clinical uncertainty while standardizing care incentivizes the development and integration of AI diagnosticians and prognosticators. In this paper, we argue that (1) responsibility gaps *are* generated by “black box” healthcare AI, (2) the presence of responsibility gaps (if unaddressed) creates serious moral problems, (3) a suitable solution is for relevant stakeholders to voluntarily *responsibilize* the gaps, taking on some moral responsibility for things they are not, strictly speaking, blameworthy for, and (4) should this solution be taken, black box healthcare AI will be permissible in the provision of healthcare.

Consider a plausible scenario involving healthcare artificial intelligence (AI): *the deferential radiologist.*

A patient has come in to have a tumor assessed. After reviewing the patient’s X-ray and collecting the relevant health information, the radiologist believes the patient’s tumor may be malignant. However, an AI technology specializing in radiology and proven to exhibit much less bias and more accurately diagnose malignancy produces a high-confidence diagnosis that the tumor is benign. The radiologist, recognizing that the AI is statistically more likely to be correct (having, perhaps, disagreed with the AI several times before and *been wrong*), defers to the AI over his own judgment and does not move forward with recommending a biopsy. The patient, as it turns out, *does* have a malignant tumor, and due to the delay in diagnosis and treatment, does not recover from the cancer and dies. Counterfactually, had the radiologist not utilized the AI, he would have stood by his conclusion about the tumor’s malignancy.

Setting aside legal questions of liability, medical error, and malpractice, who ought to be *morally blamed* for this misdiagnosis, and who should have to bear the moral fallout?^1^ Is the misdiagnosis blameworthy in the first place? It is widely thought that when AI technologies are implicated in situations that cause harm to human beings (like in our example above), this may give rise to gaps in responsibility—or responsibility gaps, for short.

The very idea of AI gives an intimation of why such gaps might arise: AI is often defined as technologies able to perform or take over tasks human beings need their natural intelligence to perform. If, ordinarily, these are tasks humans would perform and be responsible for, but they have been outsourced to technologies that are not responsible moral agents, a question arises of who, if anyone, is responsible for any problems the technologies in question cause. A gap in responsibility might be the result.

We will here focus on responsibility gaps related to what we will call “black box healthcare AI” (henceforth BBHAI) in particular. While many existing discussions of responsibility gaps within AI ethics focus on cases such as self-driving cars and military robots, our focus here is instead on BBHAI and how to deal with any gaps in responsibility that this form of AI might give rise to. The acceptability of using AI technologies is sometimes thought to partly depend on whether we can fill any responsibility gaps these technologies might create^2^—and as we see things, this applies to BBHAI just as much as it applies to other AI technologies more commonly discussed in the literature on responsibility gaps.

In this paper, we propose a resolution to responsibility gaps (henceforth R-gaps) generated by BBHAI and, on the basis of this, argue for the permissibility of their use. Drawing on the work of John Danaher, Maximilian Kiener, and Shlomo Cohen, we argue that these gaps can (and should) be willingly *responsibilized* as a form of “forced supererogation” whereby certain agents voluntarily take on responsibility for things they are not, strictly speaking, blameworthy for.^3^ Critically, our account of responsibilization is *shared* or *distributed*, distinguishing it from the typical “buck-passing” accounts which saddle a particular agent with total responsibility.

We leave it open whether our suggested solution to responsibility gaps related to medical practice and BBHAI could or should be carried over to other contexts, such as responsibility gaps related to AI used in other domains of human life. While many discussions of responsibility gaps involve an implicit or explicit assumption to the effect that all responsibility gaps, in all domains, should be resolved in the same way, our discussion here does not carry with it any such assumption.

In Section 1, we give a brief account of moral responsibility as it functions in this paper, and we also explain the general reasons typically given for why AI technologies might give rise to gaps in responsibility. In Section 2, we respond to objections that R-gaps do not exist, define four gaps we think BBHAI plausibly generate, and illustrate why those gaps are morally problematic. In Section 3, we outline shared responsibilization as a suitable strategy for dealing with BBHAI-related R-gaps and argue why clinicians, medical institutions, programmers, and their parent companies ought to responsibilize in particular ways. In Section 4, we conclude with a brief discussion on the “two horns” of contemporary R-gaps and how our approach balances competing moral interests.

## 1 Section 1: A Brief Account of Moral Responsibility and the General Idea of Responsibility Gaps

Moral responsibility is a notoriously fraught concept in the history of philosophy, but excluding skeptical arguments,^4^ most classical conceptions of human responsibility accept, at minimum, knowledge (an epistemic condition) and control (a capacity condition) as prerequisites for moral responsibility (Talbert, 2016). So, you are morally responsible for φ-ing and/or the outcomes caused by your φ-ing iff you (1) know some minimum subset of facts and/or at least believe some series of propositions about your φ-ing and (2) are able to exercise a minimum threshold of control or influence over your φ-ing. Implicitly tacked on to these conditions are additional provisos like (1a) if you do not know the minimum subset of facts and/or believe the series of propositions, your ignorance or disbelief must be inculpable to avoid moral responsibility, and (2a) if you are unable to exercise the requisite threshold of control or influence over your φ-ing, your inability must be inculpable to avoid moral responsibility.

Other conditions for responsibility have been proposed (i.e., the principle of alternative possibilities (Frankfurt, 1969), intentionality, or quality of will (Strawson, 2020), but the epistemic and control conditions will form the basis of our analysis in this paper. For our purposes, knowledge and control (as defined above) are treated as sufficient in conjunction; when taken together (and only when taken together) do they suffice for moral responsibility.

Finally, we must keep in mind that responsibility is a multi-faceted concept comprised of narrower subconcepts like culpability, answerability, accountability, and attributability, and this bears on how one thinks about responsibility gaps (Shoemaker, 2011; Watson, 1996). These distinctions will come into play later in the paper when we discuss and justify our own view, but we will bracket them for now. It might also be noted here that responsibility cuts both ways—it is not only concerned with blame and poor outcomes—but also with praise and positive outcomes.^5^ In medical contexts, as in other contexts, it can and should be asked who deserves credit for good outcomes (e.g., patients’ recovering after receiving optimal treatment). But while we think that both the positive and the negative aspects of responsibility are worthy of discussion, our focus here will be on responsibility related to bad outcomes. And we will now turn to the question of why AI technologies are sometimes thought to give rise to responsibility gaps. Why is that? What aspects of AI technologies and our relation to them are thought to create gaps in responsibility?

In order to answer these questions, we must first establish some context. Notably, the advent of powerful machine intelligence has generated several pressing ethical concerns. Domains of action and human endeavor which traditionally fell under the exclusive purview of human agents are now falling under the automated discretion of sophisticated artificial intelligence programs. Indeed, as noted above, AI is sometimes defined as the creation of technologies which can perform or take over tasks otherwise only performable by humans exercising their natural intelligence. Some contemporary examples of these forms of AI include automated medical diagnosis (Rodriguez-Ruiz et al., 2019), predictive policing (McDaniel & Pease, 2021), recidivism prediction (Dressel & Farid, 2018), cryptography (Coutinho et al., 2018), autonomous weaponry for military and law enforcement (Wyatt, 2022), and self-driving cars (Joseph & Mondal, 2022). Strong evidence suggests that, for some tasks, AI systems are already outperforming their human counterparts.^6^

Many of these AI technologies are “black box” algorithms, a designation which conveys that the AI technology’s internal mechanisms are opaque and uninterpretable to humans—even the AI technologies’ programmers (Molnar, 2019). Human operators can measure an AI’s performance at a given task, but they cannot explain *why* it behaves the way it does or *how* it arrives at its conclusions. Consider Google’s recent use of neural networks in mammogram cancer screening (The New York Times, 2020). The algorithm utilizes a form of *supervised learning*,^7^ which means that it is first fed prelabeled images (in this case, 91,000 mammograms from actual women in Britain and the U.S. whose diagnoses were already known), and the “hidden layers” of neurons work backwards, calibrating internal weights and values to try and capture the correct associations between the input data (the pixel values of X-ray images) and the prelabeled output data (cancer or no cancer). This is called the “training phase”. Next, the modelers test the AI technology against novel, unlabeled mammograms and compare its rate of false positives and negatives to human radiologists, but without any insight into *why* the AI labels a particular mammogram cancerous or not.

The concerns surrounding this kind of AI are varied,^8^ but this paper addresses a specific concern over the assignment of moral responsibility for the consequences of AI actions in the context of black box diagnostic healthcare AI (BBHAI). While much of our discussion and argument generalizes to other applications of black box AI, healthcare applications concretize a high-stakes, real-world normative context already facing mounting pressure to implement this technology.

Of particular interest in this investigation are what Andreas Matthias terms “responsibility gaps” (Matthias, 2004). Matthias’ argument, boiled down to its rudiments, looks something like the following:

1. **P1:** Artificially intelligent systems equipped with the ability to learn and optimize their performance in real-time, independent of immediate human interference or by-hand modification, make human control and prediction over their behavior very difficult if not sometimes impossible.
2. **P2:** A human being can be held morally responsible for the actions of an AI iff it can be sufficiently controlled and predicted.
3. **C:** Therefore, AIs with insufficient human control or predictive insight prevent assignment of responsibility for their actions to an appropriate human agent, creating “gaps” in responsibility.

These gaps make for real moral hazards when an AI’s actions result in harm, especially when the harm is a consequence of malfunction or error. Are such mishaps mere moral tragedies, or do they contain assignable culpability? Accepting the former will mean revising or reimagining the taxonomy of moral culpability. Automobile accidents caused by user error, for instance, are ordinarily blamed on the driver, but if the driver is not a person, is the accident an unattributable misfortune? Many contributors to the field of ethics find this conclusion unpalatable, if not implausible, because it carries the unsavory implication that victims of AI decision-making and action have no moral right to restitution or recompense when they have been harmed.^9^ To avoid these threats, we must find suitable recipients of blame and responsibility, both prospectively (for preempting AI mistakes) or retroactively (when somebody is being held accountable after the fact).

With these general comments about responsibility gaps in place, let us now turn to our particular focus in this paper, namely, responsibility gaps related to black box healthcare AI. As we discuss this, we will also discuss the points of view of some authors who have recently argued that we should be skeptical about the idea of responsibility gaps created by AI technologies. Considering some such views will help to clarify why black box healthcare technologies creates responsibility gaps that we need to be concerned about.

## 2 Section 2: Possible BBHAI Gaps and Possible Strategies for Filling the Gaps

### 2.1 Do Black Box Healthcare AI Generate Responsibility Gaps?

While much of the debate surrounds *what to do* about R-gaps, an approximately equal amount of the debate concerns whether R-gaps even exist in the first place.^10^ We have dubbed “*responsibility gap deniers*”—or “*deniers*”, for short—those who reject the idea that black box AI create gaps in assigning responsibility. Accordingly, arguments like our own which treat the existence of R-gaps as a first premise will not get off the ground without addressing the claims of deniers. Generally, deniers make one of two claims:

- **Appeals to normative standards**: AI actions and their consequences do not generate any responsibility or responsibility gaps because, according to certain normative standards (e.g., standard of care), no wrongdoing has occurred.
- **Tracing or buck-passing**: AI actions and their consequences can be traced back through an agential chain of causality to some appropriate stopping point, so there *is* ultimately a source of responsibility.^11^

The mileage of appeals to normative standards will vary by the AI’s use domain. A common refrain in industry is the appeal to acceptable quality limits (AQL) used in quality control. AQL is a statistical metric and standard for the maximum acceptable number of defective goods allowed in a particular sample size. So, a factory producing widgets might be permitted by its supplier to have 2.5% defective widgets for every batch.^12^ The FDA likewise regulates AQLs in clinical goods like surgical masks and gloves (Food & Drugs, 2022). If AI developers produce a BBHAI that correctly diagnoses cancer 97.5% of the time, it could then be said that the 2.5% margin of error meets a normative standard for tolerable risk of misdiagnosis.^13^

Another appeal unique to the clinical setting is the standard of care; if a machine learning algorithm has been properly validated, tested in situ, proven to improve clinical outcomes with more reliable predictions, and is widely accepted as the standard of care, then the physician’s choice to defer to the AI should not be considered a wrong even if it results in a patient’s being harmed. Finally, with respect to the black box element, some deniers argue that opacity is not new to clinical decision-making. Alex London points out that the “clinical gestalt” by which a clinician holistically evaluates a patient’s condition is not often very transparent or well-documented, and neither are the causal mechanisms of medical interventions we routinely rely on, like certain prescription medications (London, 2019). *Ceteris paribus*, if we consider the standards of care of modern medicine permissible, and BBHAI reflect those standards, then BBHAI are likewise permissible, and *there is no R-gap.*

Whereas appeals to normative standards accounts deny that every moral responsibility needs a bearer, tracing and buck-passing accounts accept the need for an assessment and attribution of moral responsibility but deny the inception of any gaps.^14^ Tracers tend to locate responsibility either with the AI’s end-user or the AI’s programmers. Ezio Di Nucci, for instance, argues that we can trace the responsibility to whichever agent ultimately chooses to delegate a given task to the AI technology in question (Di Nucci, 2021). If, for example, clinicians in a hospital utilize an AI technology to randomize allocation of scarce hospital resources according to an equity-weighted lottery, they are equally as responsible for the resulting allocation decision as if they personally throw darts at an equity-weighted dart board. In this case, the automation of an action one would have otherwise taken does not seem to exculpate whoever initiates the automated process. Put another way, “the delegator does not lose any responsibility for the outcomes achieved by the delegatee” (Danaher, 2022: 26). In *deferential radiologist*, the radiologist chose to utilize and defer to the AI, and so it might be thought that the responsibility-buck ought to stop with him.

Tracing or buck-passing accounts are frequently justified on the grounds of some role a human agent is alleged to have in relation to the BBHAI, or the duties incumbent on the agent rooted in a profession or title. Implicit in these arguments is an antecedent argument that AI actions and their consequences are or ought to be anchored to “humans in the loop,” who are responsible by virtue of their supervisory or collaborative relationship to the AI. This intuition is shared by many, as so-called “centaur” models of AI implementation emphasize the importance and benefits of pairing humans and AI together and keeping the human operator in a responsibility-bearing position.^15,16^ The US military’s Defense Science Board gestures to this point incisively in a 2012 report about AI weapons systems:

… there are no fully autonomous systems just as there are no fully autonomous soldiers, sailors, airmen, or Marines… Perhaps the most important message for commanders is that all machines are supervised by humans to some degree, and the best capabilities result from the coordination and collaboration of humans and machines. (U.S. Department of Defense Science Board, 2012)

Applied to the *deferential radiologist* case, one might argue either that (1) the radiologist failed some duty as a collaborator or supervisor by deferring to the AI instead of seeking further clinical consultation or recommending the biopsy just in case, and this justifies their responsibility, or (2) simply by virtue of existing in a supervisory or collaborative capacity as a diagnostician with the BBHAI, the radiologist takes on any accompanying responsibility.

None of these debunking arguments are without merit, but they fail in plugging or side-stepping the R-gap. Appeals to normative standards accounts effectively question-beg insofar as they take for granted that the clinical standard of care is sufficient for moral permissibility or that the purported opacity of physician judgment already present in clinical practice is unproblematic. They succeed not in disproving R-gaps, but in testing discussants’ moral intuitions for inconsistency and highlighting potential double-standards for AI integration.^17^

With respect to tracing, *causal* responsibility seems a necessary but insufficient condition for *moral* responsibility, and in any case, the purely causal approach seems muddied by agentially diffuse chains of causal interdependence whereby many agents seem to contribute to the outcome, not just the solitary end-user or original programmer. In this way, our *deferential radiologist* case is an oversimplification of actual clinical practice, where specialized committees and medical review boards comprised of many experts convene to reach a decision on high-impact or high-risk care decisions. Moreover, unlike Di Nucci’s example of the automated resource allocator, the agent in *deferential radiologist* is not merely offloading the labor (randomization) for a decision already made (to allocate resources according to equity-weighted chance) and claiming a clean conscience (what Rubel et al. call “agency laundering”) (Rubel et al., 2019). The radiologist is instead actively mediating his own epistemic standing with that of the AI *in order to decide what to do*, which suggests that it is the collaborative aspect which holds the best odds of establishing the radiologist’s moral responsibility for the misdiagnosis.^18^

To reiterate, accepting the existence of R-gaps does not mean exculpating the human agent(s) of all possible responsibility—only the responsibility represented by the gaps. The radiologist may bear all the ordinary moral responsibility which falls outside the gaps, and some of this may relate to their duties as a collaborator (which we elaborate on in the following section). That said, it is strange to suggest that active collaboration between a human and AI establishes why the human should bear the totality of responsibility when it seems it is precisely owing to the relationship being *collaborative* that the human ought *not* to receive full blame.^19^

It seems to be a consequence only of the fact that the AI is, *per definitionem*, intrinsically inculpable and therefore not a fitting subject of responsibility that, by process of elimination, the human should bear responsibility for their joint problem-solving and any resultant consequences. Understood in this way, R-gaps are an emergent phenomenon of the nebulous interspace between a human’s agency and the agency-like properties an AI possesses—properties like autonomous action, independent reasoning and learning, and the epistemic authority to inform or contribute to decision-making.

So, what kinds of responsibility gaps do BBHAI potentially generate, and are they a problem? As one of us has pointed out elsewhere, responsibility can be both forward-looking (prospective) and backward-looking (retrospective), and this allows us to tease apart different species of responsibility gaps.^20,21^ Utilizing this distinction, we propose two forward-looking and one backward-looking R-gap, and one mixed case of a both backward and forward-looking gap plausibly generated by BBHAI and in our *deferential radiologist case*:

1. **Anticipatory Gaps:** Forward-looking gaps related to responsibility for anticipating when, where, or for whom a black box diagnostic tool’s margin of error will manifest and appropriately preempting any resultant medical error.
2. **Explanatory Gaps:** Forward-looking gaps related to responsibility for explaining to a patient, caregiver, or family member *how* or *why* a black box tool arrives at its prognosis and/or recommendations.
3. **Retrospective Accountability Gaps:** Backward-looking gaps related to responsibility for harmful outcomes resulting from black box prognoses and/or recommendations after the fact. Broadly construed, this can include compensatory, legislative, or interpersonal actions aimed at taking responsibility for past harms.
4. **Corrective Gaps:** Both forward-looking and backward-looking gaps related to responsibility for preventing future recurrences of an error which has already occurred in the past.

These gaps can be identified by applying the “ought-implies-can principle,” where asserting that an agent *ought* to φ necessarily implies that it is *possible* for them to φ. As discussed in Section 2, we should think of “possibility” in this context as constituting an epistemic condition and a control condition working in tandem. Ordinarily in healthcare, we think clinicians *ought* to take actions to preempt mistakes, explain how they come to a diagnosis, account retrospectively for harm caused by their errors, and take positive steps to prevent future recurrences of the error in question. What is important is that these obligations can, in principle, be fulfilled by clinicians. When a BBHAI is introduced to the equation, however, it becomes unclear whether it is possible to fulfill these duties, and consequently, responsibility gaps are potentially created.

R-gaps represent a real moral danger in healthcare because they interfere with clinicians’ ability to fulfill moral duties and uphold patients’ rights. For example, as Ryan Felder points out, what we call the Explanatory Gap can violate a patient’s right to informed consent if informed consent is held to include explanations upon request of how/why a clinical team is coming to a diagnosis or making treatment recommendations (Felder, 2021). Or take, for instance, the Corrective Gap. One of us has argued elsewhere that a crucial step for any medical community that has erred is admitting failure, pledging to do better, and ensuring that whatever harm dealt was not suffered in vain.^22,23^ Given that the AI is a black box, however, it is not possible to determine what caused it to misdiagnose a particular mammogram, nor to explicitly instruct it in ways that it will not misdiagnose similar patients in the future. With these R-gap candidates and their costs established, we move now to the resolution we propose: shared responsibilization.

## 3 Section 3: A Shared Responsibilization Approach

*Responsibilization* is a school of responses to the R-gap described by John Danaher in which individuals *take upon themselves* the responsibility generated by the R-gap:

Responsibilisation is where we confront the tragic choice head-on and accept responsibility for resolving the conflict in a particular way. We do not pass the buck to someone else; we do not bury our heads in the sand. We embrace the fact that life sometimes involves these tragic tradeoffs, and we live with the consequences. We accept that it is our moral responsibility to decide. (Danaher, 2022: 25)

There are two things to note about Danaher’s project and our use of his terminology. First, Danaher is discussing R-gaps in the context of “tragic moral choices” for which we have competing moral imperatives and any choice one makes will feel morally costly. We extend responsibilization to include cases of choice under ineliminable, epistemic aporia (like that of *deferential radiologist*) where the outcomes may be tragic or morally costly, but the choice itself is not. Secondly, it should be clarified how a responsibilizer differs from the denier class of replies. Deniers assert either (1) there is no responsibility in need of assigning (appeals to normative standards) or (2) there are no R-gaps because conventional approaches to responsibility succeed in assigning the responsibility generated to a rightful owner (tracing and collaborative/supervisory arguments). Responsibilizers, by contrast, recognize an irreducible *moral remainder* out in no-man’s land and opt, for various reasons, to bear it anyways.

What we propose is a version of the *responsibilization* approach to R-gaps which is *shared*. By shared, we mean that various key stakeholders ought to collectively responsibilize (viz., assume responsibility for) the gaps. In the case of BBHAI, the medical institution and its staff implementing the device ought to responsibilize the Explanatory and Retrospective Accountability gaps, and the modelers, data scientists, and their parent companies ought to responsibilize the Corrective and Anticipatory gaps. (We note that this type of suggestion may be less plausible in other domains, in relation other forms of AI used within other forms contexts—e.g., like self-driving cars. It may, however, also work in other contexts that involve clear organizational structures, such as those related to AI used in military contexts or law-enforcement contexts.)

We anticipate several questions about this proposal. How does one responsibilize? Why should someone who is not blameworthy responsibilize? And what would responsibilizing entail for the BBHAI R-gaps? To the first question, we draw on Maximilian Kiener’s argument that it is possible to exercise a normative power to take on the otherwise unassigned responsibility of R-gaps (Kiener, 2022). What we call *mea culpa speech acts* can have the illocutionary effect of transferring or taking on responsibility. For instance, saying “it was my fault” can be both an honest avowal of fault, but also a form of capitulation or dispute-settling by offering to take the blame absent conviction that one deserves it, or amid irresolvable ambiguity over who is at fault. (This, again, may work better in some contexts than others.)

This kind of moral cross-bearing might ordinarily be considered a form of supererogation, but as it relates to R-gaps, it is a perfect fit for what Shlomo Cohen calls “forced supererogation” (Cohen, 2015). By forced, Cohen does not mean that the act is compulsory (morally or otherwise), but rather that the agent is in the position of a forced choice between either a supererogatory act, or a wrongful but blameless act. This is distinct from ordinary supererogation, where the agent retains a choice representing moral mediocrity or merely permissible action in between praiseworthy and blameworthy acts. Thus, cases of forced supererogation must exhibit three parameters: “(1) performing the commendable action is especially praiseworthy; (2) not performing is not blameworthy; and (3) not performing is wrong.”^24^

An objection may follow that there is a healthy middle ground between responsibilizing and complete delegation or agency laundering in the case of medical BBHAI. A clinician could express compassion and empathy for a patient’s plight at having been harmed without responsibilizing the BBHAI’s error. After all, a patient might interpret a clinician’s mea culpa speech act as admittance of wrongdoing and grounds for litigation, and so it could be safer for the clinician to opt instead for merely empathizing with the patient and meaningfully engaging with them about the harm dealt. This approach of responding to harm dealt by BBHAI-informed care has the demerit of ignoring the R-gap. Moreover, even if this supposition about mea culpa speech acts inviting litigation were true, it is not clear that, on balance, clinicians would be worse off. If a clinician relies on a tool which, in aggregate, improves diagnostic accuracy and quality of care, then even in terms of liability, one should expect a net decrease in medical error compared to continuing a practice solely reliant on human judgment.^25^

Voluntarily responsibilizing is also especially praiseworthy for several reasons. First, it makes the use of BBHAI permissible (and BBHAI technologies improve clinical care and promise substantial aggregative benefits). Second, it ensures that those harmed by BBHAI have some recourse to redress. Third, it reflects a self-sacrificing virtue whereby the agent prioritizes the benefits of BBHAI to others over the moral risk it poses to themself. Failing to responsibilize is, of course, blameless for the reasons already covered at length in this paper, so it remains for us to try to demonstrate why it is nonetheless wrong, as well as why physicians, medical institutions, programmers and their companies, all ought to responsibilize.

### 3.1 The Voluntariness of the Epistemic Position and the Vice of Failing to Responsibilize

As noted in Section 2, responsibility has an epistemic condition on which, in order to be responsible for doing X or preventing Y, you must either *know* or *be culpable for knowing* certain facts necessary to do X or prevent Y. When it comes to the four gaps we have proposed, the opacity of BBHAI seems to make knowledge of the requisite facts impossible. Ignorance, however, does not always forestall blame or responsibility, because not all ignorance is excusable. Holly Smith has coined the term “benighting act” to describe acts which culpably hinder or impair one’s epistemic position. For our purposes, we might ask whether the radiologist in *deferential radiologist* (or any of the other agents in the context of BBHAI) have committed benighting acts. (Smith, 1983).

It is our position that the very creation and existence of a BBHAI has, as an ineluctable effect, mutually assured benightedness. By implementing a BBHAI, one invites a kind of epistemic impoverishment in the form of reduced insight into the basis for clinical diagnoses and recommendations, as well as the nature of possible errors therein. Conversely, by neglecting to utilize a BBHAI, one remains in the proverbial darkness of human fallibility, bias, and cognitive ceilings. In effect, by opening one eye, you close the other. No matter what you choose, you will be culpably ignorant of *something*. Clinicians and medical institutions are thus not responsible for the BBHAI being uninterpretable, unpredictable, and irremediable, or for what the BBHAI outputs. What they *are* responsible for is entering into a position of voluntary epistemic blindness by choosing to implement and rely on the BBHAI. As we see things, this is the first reason why failing to responsibilize is a moral wrong: R-gaps are collectively and informedly consented to. They represent a *foreseen but unintended* consequence of using BBHAI, but they are still consequences which are *chosen* and *accepted*.^26^

One might object that this places those like the radiologist in an unfair double bind. On the one hand, assuming sufficient accuracy, the radiologist is arguably obligated to defer to the BBHAI and neglecting to do so would be a moral failing. In exchange for taking correct action, however, the radiologist is then punished by bearing R-gap costs.

Several replies are in order. First, as Cohen argues, part of the harsh reality of forced supererogation cases is that they “defy the commonsensical expectation that people ought to have the option not to be moral heroes without being in the wrong” (Cohen, 2015), and this is intractable. Second, this cost is significantly mitigated by the shared responsibilization proposal we are advocating. No individual should be “taking the rap” all by themselves. Returning to epistemic voluntariness, *all* relevantly involved agents know (or are culpable for knowing) about R-gaps and of the ex-ante margin of error the AI poses.

We readily concede that it would be quite unfair for all the ex-post costs to be meted out to one person on the grounds of moral (un)luck. Instead, given that collectively, the programmers and parent company design a BBHAI knowing it will create R-gaps, and the medical institution and its clinical staff purchase and implement the device knowing it has R-gaps, they should all *share* in responsibilizing the gaps. From there, it is a simple matter of divvying the gaps up accordingly.

Ordinarily, programmers (and their supporting institutions) are morally responsible for the defects in their AI—for avoiding known bugs, troubleshooting errors and patching the AI via software updates (Corrective and Anticipatory gaps). These gaps are most proximal and fitting to programmers and their parent companies, just as ensuring informed consent (Explanatory gap) and taking ownership for past medical errors (Retroactive Accountability Gap) are most proximal and fitting to clinicians and medical institutions. Another reason for assigning the gaps this way is because it reflects the kinds of virtues arguably most desirable in each respective party.^27^ We want programmers and their parent companies to be meticulous about what they create and conscientious about how it will impact others and how it might be used (i.e., dual-use considerations). Feasibility constraints notwithstanding, programmers and their parent companies ought to be invested in improving their product when its shortcomings have moral costs. Programmers and their parent companies refusing to responsibilize are liable to cultivate cavalier, dismissive, or callous attitudes toward the users and potential victims of their technology. Likewise, we want clinicians and medical institutions to acknowledge the imperfections of their provision of healthcare and address patients and their families honestly, directly, and sincerely in the event of medical harm. Clinicians and medical institutions failing to responsibilize are liable to become complacent, treating clinical decision-making as a perfunctory rubberstamp on AI recommendations and disengaging from the clinician-patient relationship built on mutual trust and transparency.

### 3.2 So What Does Shared Responsibilization Entail for BBHAI?

We do not have the space nor the expertise to provide a comprehensive blueprint of how shared responsibilization might be codified in law or policy, but we can outline how we see it being enacted by individual agents. For the radiologist, it might look like saying to the patient: “Relying on technology which rarely makes mistakes, I did not recommend you receive a biopsy, and that has caused you grave harm. I believed I was sparing you an invasive surgery, but in fact I imparted a false sense of security. It is my responsibility as your doctor to ensure your health and wellbeing, and I am sorry to have failed in that charge.” For the medical institution, they perhaps ought to have a hand in recouping the financial/medical losses incurred by patients and their families even if they do not consider BBHAI error to be a form of medical malpractice. Finally, the modelers and AI development companies ought to continually revise and study the AI they sell or deploy—using its errors to recalibrate its weights and improve accuracy, even if incrementally. The ways in which one could enact or take up responsibility will vary by context and stakeholder, and what we have outlined is only one possible model.

In all these cases, the lodestar virtue is *answerability* for one’s role, profession, and choices—a virtue obfuscated by preoccupation with one’s own individual blamelessness and insistent exculpation along the lines of: “well the AI we built is just *like that* and messes up sometimes” or “the AI got the diagnosis wrong, so it’s not my fault.” It is true you are not to blame, but that does not mean you are any less implicated in the moral stakes at hand.

## 4 Conclusion: The Two Horns of the Responsibility Gap

As Matthias points out in his original paper, *we need* AI with the ability to learn in real time, rout out human bias, and see things in ways humans cannot (“computer vision”) (Matthias, 2004). We take it as highly plausible that the greater the degree to which a BBHAI outperforms human operators and mitigates human error, the greater the moral imperative to collaborate with or (to the extent it outperforms the human) defer to the BBHAI.^28^ The costs of rejecting or dismissing AI outright, therefore, cannot be overstated, nor the potential windfall of utility promised by continued development under responsible and equitable stewardship. Moreover, the genie is well and truly out of the bottle; AI is here to stay, and so any R-gap purist with ambitions of avoiding R-gaps entirely will soon find the world morally unnavigable.

Thus, we are left with the two horns of the R-gap. On one horn, if we opt out of gap-generating tech, we miss out on potentially epoch-defining boons for human wellbeing. On the other horn, if we opt in, we must either find ways to either reconcile R-gaps with our traditional normative landscape or get to work redefining the normative landscape to be more compatible with the technology at hand.

In response to this dilemma, we have provided an account on which R-gaps can be responsibilized to those with a hand in designing and/or utilizing the technology itself. By classifying this responsibilization as a kind of forced supererogation, we give due consideration to the blamelessness of the parties involved, the foreseen-but-unintended nature of R-gaps, and the correctness of their collective choices (to develop and implement BBHAI), as well as the importance of cultivating virtuous ideals and the danger of eroding agency and answerability for professional roles and decision-making.

Again, we suggest this strategy with respect to how to fill responsibility gaps related to BBHAI in medical contexts. Whether this sort of approach is also suitable for other contexts in which AI technologies are used and where responsibility gaps may arise, we view as an open question worthy of consideration.

## Data Availability

This is a theoretical paper. No data was used in this research.

## Footnotes

1 The legal implications of introducing AI to medicine are varied and beyond the scope of this paper. For some helpful approaches to the legal aspects of healthcare AI, see Price et al. (2019) and Gerke et al. (2020).

2 See, e.g., List (2021), in which List argues that we should only tolerate new forms of AI technologies if we are able to fill any potential responsibility gaps they might give rise to. For a contrary argument to the effect that we can tolerate responsibility gaps if the overall benefits of the technologies are great enough—presented in the context of military AI—see Simpson and Muller (2016). We follow List’s lead in thinking that whether we can fill responsibility gaps is a key consideration of whether novel AI technologies are morally permissible.

3 “Forced supererogation” may sound oxymoronic at first blush given that supererogation generally refers to moral acts “beyond the call of duty.” The concept, borrowed from Schlomo Cohen and explained in greater detail later in this paper, refers to a circumstance in which an agent must either perform a supererogatory act or a blameless but nonetheless wrong action. See Cohen (2015).

4 For moral luck arguments, see. Williams and Nagel (1976); for incompatibilist arguments, see Pereboom (2014); for regress/ultimate responsibility arguments, see Strawson (1994).

5 Nyholm (2023a, b). See also Danaher and Nyholm (2021).

6 The mammogram AI discussed in the following paragraph, for instance, has demonstrated a 9.4% reduction in false negatives and a 5.7% reduction in false positives, relative to the standard margin of error among US radiologists. See The New York Times (2020).

7 For more detail on supervised and unsupervised learning, see Russell et al. (2010).

8 Ranging from automation-driven unemployment (and loss of human meaning derived from work and achievement) to algorithmic bias worsening existing patterns of social injustice, this latter problem is compounded by automation bias lending unearned credence to algorithms and the biases being nested within a black box and, thus, difficult to pin down or notice.

9 Again, see List (2021) for one example of a paper defending such a stance viz. R-gaps.

10 See, for example, Tigard (2021), Bryson et al. (2017), and Königs (2022).

11 Santoni de Sio and van den Hoven do not only talk about “tracing” in this context but also “tracking”—they argue that if AI technologies behave in a way that tracks human interests and we can trace their behavior back to human beings who understand how they work and their moral significance, then these AI technologies are under “meaningful human control,” and this helps to make sure that there are no responsibility gaps. See Santoni de Sio and van den Hoven (2018). For critical discussion, see Nyholm (2023b).

12 You might consider this a normative and not merely legal or market-driven standard in circumstances where pushing the margin of defective product to 0% may be prohibitively costly, technologically infeasible, or humanly impossible. Requiring companies to have perfect quality assurance might require them to downsize their operations and reduce the number of people able to receive the service they provide, which might be a net loss in terms of human benefit.

13 Similar arguments might be made about extremely rare allergic reactions to certain medications or vaccinations. A friend of one of the authors once had a brain tumor removed, and after the surgery, they needed to be certain of sufficient vascular activity and so used contrasting dye for the fMRI. The dye caused an anaphylactic reaction and the mother needed to be intubated. Extreme reactions to this dye (Lymphazurin) have been observed in only 1–3% of patients according to one study (see Liang & Carson, 2008).

14 See Tigard (2021).

15 Nyholm (2020): chapter three. See also Nyholm (2023a): chapter six.

16 Indeed, during testing of the Google mammogram BBHAI, while the algorithm outperformed the US radiologists on average, there were still cases where the algorithm missed a cancer that *all six radiologists* found, suggesting that synthesis of human and AI judgments is often the optimal strategy.

17 See Zerilli et al. (2019) and Kempt et al. (2022).

18 For more discussion of the idea of uses of AI technologies as a form of human–machine collaboration, see Nyholm (2018).

19 An objection may follow that the radiologist is failing some collaborative duty. Maybe the radiologist failed to adequately resolve the disagreement between himself and the BBHAI—maybe he was too quick to surrender his suspicions. This objection lacks force. If the radiologist knew that the BBHAI was more accurate than himself, and if he had no recourse to get to the root of their difference of opinion, what should we expect of him? It is scenarios like this that motivate some to argue that BBHAI should serve only as confirmatory tools such that disagreement triggers consultation with another human to tie-break the dispute. As one of us has written elsewhere, while this may work as a band aid solution, it will not scale with the ever-growing power of AI models. The rule of thumb whereby AI-human disputes are resolved by just letting more humans weigh in may soon prove an anachronistic epistemic standard, not least because the very human biases and fallibilities which lead the first human to err may cause subsequent humans to similarly fall short. See Lang (2022).

20 Santoni de Sio and van den Hoven do not only talk about “tracing” in this context but also “tracking”—they argue that if AI technologies behave in a way that tracks human interests and we can trace their behavior back to human beings who understand how they work and their moral significance, then these AI technologies are under “meaningful human control,” and this helps to make sure that there are no responsibility gaps. See Santoni de Sio and van den Hoven (2018). For critical discussion, see Nyholm (2023b).

21 See, Nyholm (2023a, b). There are different possible ways of classifying different R-gaps. The just-mentioned book chapter distinguishes among responsibility gaps that are backward-looking and forward-looking, as well as between responsibility-gaps that are positive (what might be called praise gaps) and negative (what might be called blame gaps). For another classification of different types of responsibility gaps, see Santoni de Sio and Mecacci (2021). Their categorizations concern, among other things, differences between gaps related to individual culpability, and gaps related to accountability on the part of public officials.

22 See Lang (2021).

23 One might think the Corrective Gap is more appropriately characterized as primarily being a forward-looking gap given that it stems from an inability to take *future* actions to prevent BBHAI error. However, we think that the best way of looking at it is a mixed case, with both backward-looking and forward-looking elements. The backward-looking element relates to who is responsible for a past harm. The forward-looking element of this idea relates to who is responsible for corrective measures in relation to potential future harms. This can be compared and contrasted with the Anticipatory gap in the following way. Whereas the Anticipatory Gap represents a duty primarily owed to future persons, the Corrective Gap represents a duty that is also importantly owed to past persons as a way of taking responsibility for past events. When in 2012, a hospital located in New York City erroneously discharged a 12-year-old boy who shortly thereafter died of sepsis, part of the hospital’s response was to institute a new discharge policy and require additional checklists be filled out before discharging a patient. This change was expected to reduce future loss of life, yes, but it was also a form of rectification *owed to the victim and his family*. The policy is aimed at ensuring that his fate would not be suffered by others—that his death and legacy would positively contribute to the safety of those who came after. See Dwyer (2012).

24 ibid, 1008.

25 We thank a reviewer for suggesting this possible objection to our view.

26 Classic “doctrine of double effect” cases might have application here. Perhaps it is not wrong for me to pull the trolley lever which kills the bystander, *but I still did it*—the consequence is still mine.

27 For discussion of how to relate virtue and responsiblity-taking, see Van de Poel’s chapter “Moral Responsiblity” in (Van de Poel et al., 2015).

28 This can be compared to the idea sometimes discussed in relation to self-driving cars according to which, if self-driving cars eventually become much safer than regular cars, the greater the moral imperative to use—and perhaps switch over to only using—self-driving cars (as opposed to regular, potentially less safe cars). See for example Sparrow and Howard (2017) and Nyholm (2020): chapter four.
